# Development of Thermoplastic Bi-Component Electrodes for Triboelectric Impact Detection in Smart Textile Applications

**DOI:** 10.3390/polym17020210

**Published:** 2025-01-16

**Authors:** David Seixas Esteves, Amanda Melo, Bruno Peliteiro, Nelson Durães, Maria C. Paiva, Elsa W. Sequeiros

**Affiliations:** 1 Department of Mechanical Engineering, Faculty of Engineering, University of Porto, 4200-465 Porto, Portugal; ews@fe.up.pt; 2 CeNTI, Centre for Nanotechnology and Advanced Materials, 4760-034 Vila Nova de Famalicão, Portugal; amelo@centi.pt (A.M.); bpeliteiro@centi.pt (B.P.); nduraes@centi.pt (N.D.); 3 Department of Polymer Engineering, IPC—Institute for Polymers and Composites, University of Minho, 4800-058 Guimarães, Portugal; mcpaiva@dep.uminho.pt; 4 LAETA/INEGI–Institute of Science and Innovation in Mechanical and Industrial Engineering, 4200-465 Porto, Portugal

**Keywords:** MWCNT, polymer composites, smart textiles, sensors, advanced materials

## Abstract

Smart textiles provide a significant technological advancement, but their development must balance traditional textile properties with electronic features. To address this challenge, this study introduces a flexible, electrically conductive composite material that can be fabricated using a continuous bi-component extrusion process, making it ideal for sensor electrodes. The primary aim was to create a composite for the filament’s core, combining multi-walled carbon nanotubes (MWCNTs), polypropylene (PP), and thermoplastic elastomer (TPE), optimised for conductivity and flexibility. This blend, suitable for bi-component extrusion processes, exemplifies the role of advanced materials in combining electrical conductivity, mechanical flexibility, and processability, which are essential for wearable technology. The composite optimisation balanced MWCNT (2.5, 5, 7.5, and 10 wt.%) and TPE (0, 25, and 50 wt.%) in a PP matrix. There was a significant decrease in electrical resistivity between 2.5 and 5 wt.% MWCNT, with electrical resistivity ranging from (7.64 ± 4.03)10^4^ to (1.15 ± 0.10)10^−1^ Ω·m. Combining the composite with 25 wt.% TPE improved the flexibility, while with 50 wt.% TPE decreased tensile strength and hindered the masterbatch pelletising process. The final stage involved laminating the composite filament electrodes, with a 5 wt.% MWCNT/PP/(25 wt.% TPE) core and a TPE sheath, into a textile triboelectric impact detection sensor. This sensor, responding to contact and separation, produced an output voltage of approximately 5 V peak-to-peak per filament and 15 V peak-to-peak with five filaments under a 100 N force over 78.54 cm^2^. This preliminary study demonstrates an innovative approach to enhance the flexibility of conductive materials for smart textile applications, enabling the development of triboelectric sensor electrodes with potential applications in impact detection, fall monitoring, and motion tracking.

## 1. Introduction

Smart textiles offer responsive capabilities to external stimuli such as thermal, mechanical, chemical, electrical, magnetic, or optical changes. These materials interact intelligently with their surroundings, adapting to environmental changes with remarkable versatility [1,2,3]. The evolution of smart textiles has led to three distinct generations: passive smart textiles (first generation) that monitor environmental changes through sensors; active smart textiles (second generation) that both sense and respond to stimuli; and highly intelligent textiles (third generation) that can react to and adapt to environmental conditions autonomously [4]. Smart textiles can be integrated into products using different methods, including weaving, knitting, embroidery, lamination, and braiding [4,5,6,7]. These integrated smart textiles have demonstrated transformative potential across diverse sectors such as healthcare, sports, automotive, aerospace, and military [8,9]. For example, in healthcare, wearable monitoring systems measure vital signs and perform gait analysis through capacitive or triboelectric sensing [10,11,12,13], while in the automotive and aerospace sectors, embedded sensor systems enhance safety [14,15].

Central to smart textile development is the selection of appropriate conductive materials [7,16,17]. Multi-walled carbon nanotubes (MWCNTs) have emerged as a particularly promising option, excelling in applications ranging from thermal regulation to electromagnetic shielding [18,19,20,21]. Their exceptional electrical conductivity and versatility have made MWCNT-based composites fundamental to sensor technologies, including piezoelectric, triboelectric, and thermoelectric devices [22,23,24,25]. These sensors have proven especially valuable in wearable technology and remote monitoring systems [26,27,28,29].

Within the field of passive smart textiles, triboelectric impact detection sensors have become increasingly necessary for real-time monitoring across various sectors. Healthcare applications include fall detection systems for elderly care [30,31], whilst aerospace implementations focus on damage detection from debris or collisions [32]. The effectiveness of these sensors depends on carefully calibrated operational parameters, including activation force thresholds and environmental condition tolerances [33].

This study advances the field of smart textiles by introducing a novel approach for developing MWCNT-based triboelectric sensor electrodes, designed for continuous production and direct lamination within textile structures. By using optimised electrically conductive and flexible polymer composites, processed via bi-component extrusion melt spinning, it is possible to overcome some of the development limitations of conventional triboelectric devices, such as poor mechanical robustness, limited flexibility, insufficient environmental stability, and complex manufacturing processes [34,35]. Furthermore, this innovative approach provides a balanced solution, integrating a conductive core with a thermoplastic elastomer (TPE) sheath that is compatible with low-temperature textile lamination processes, to ensure reliable adhesion for electrode integration.

## 2. Materials and Methods

The experimental process, outlined in Figure S1 (Supplementary Materials), begins with the analysis of the electrical, mechanical, rheological, and thermal properties of polymer tapes containing varying weight percentages of MWCNTs. To further enhance the mechanical properties of these composites, TPE was blended with polypropylene (PP). Once the optimal composite samples were identified, they were processed through a bi-component extrusion technique to produce sheath/core structured filaments. These filaments were subsequently laminated onto textile substrates, serving as electrodes for the fabrication of triboelectric sensors.

### 2.1. Materials

MWCNTs were used as a PP masterbatch, containing approximately 20 wt.% of MWCNTs, supplied by Nanocyl (PLASTICYL PP2001, Sambreville, Belgium). PP was bought from Repsol (ISPLEN PP 086, Madrid, Spain), while the TPE used was Vistamaxx 6202, supplied by ExxonMobil (Irving, TX, USA). The polymer compatibilizer, Exxelor, was sourced from The Compound Company (Enschede, The Netherlands).

### 2.2. Tape Fabrication Process

Composites of PP/MWCNT were produced in a range of MWCNT concentrations aiming at electrical conductivity, from the early stages of electrical percolation to higher conductivity composites, based on previous experience and the literature [3,36,37]. Thus, the weight %s of 2.5, 5, 7.5, and 10 were selected for the PP composites preparation.

Seven distinct composites were synthesised using a twin-screw extruder to form and characterise the tapes. MWCNT masterbatch (PLASTICYL PP2001) with a nominal content of 20 wt.% MWCNTs in PP was utilised by dilution to produce the different composites. The masterbatch was initially diluted with PP (ISPLEN PP 086) to achieve MWCNT concentrations of 2.5, 5, 7.5, and 10 wt.% within the PP matrix. Subsequently, to enhance polymer flexibility, samples containing 5, 7.5, and 10 wt.% MWCNTs in PP were blended with 25 wt.% TPE (Vistamaxx 6202). A final composition with 10 wt.% MWCNTs and 50 wt.% TPE was also prepared by diluting the 20 wt.% MWCNT/PP masterbatch. Additionally, 2.5 wt.% of Exxelor PO 1020 was added to all samples to improve polymer compatibility.

The tapes were extruded using a co-rotating twin-screw extruder with L/D 25 (Rondol Technology Ltd., Staffordshire, UK) under specific conditions: temperature along the extruder was set at 200, 210, 215, 215, and 220 °C for screw zones 1 through 4 and the die, respectively. The drive torque was maintained at 40%, and the screw speed was 100 rpm. Extrusion was conducted using a flat die of 19 mm width and 0.7 mm thickness, and the tapes were collected using pulling rolls at constant speed.

### 2.3. Cross-Section Morphology Analysis

Morphological analysis for the visualisation of the MWCNT distribution on the cross-section of the polymeric tapes was conducted using a Field Emission Gun Scanning Electron Microscope (FEG-SEM, NOVA 200 Nano SEM, FEI Company, Hillsboro, OR, USA) in secondary electrons (SE) mode. Before analysis, specimens were cryo-fractured using liquid nitrogen and coated with a 1.5 nm film of Au-Pd (80–20 weight %) using a high-resolution sputter coater (208HR, Cressington Company, Watford, UK), coupled with an MTM-20 High Resolution Thickness Controller (Cressington).

### 2.4. Mechanical Properties

Tensile tests of the tapes were carried out to evaluate the impact of MWCNTs and TPE on the mechanical properties of the composites. The tests were conducted on a Shimadzu AGX-V (Shimadzu Corporation, Kyoto, Japan) equipped with a 5 kN load cell and rubber-coated grips. The geometry of the test specimens was rectangular with 100 mm length, 10 mm wide and approximately 0.5 mm thickness, with a gauge length (L0) of 30 mm. The tests were performed at a speed of 1000 mm/min and repeated three times for each composite. The 1000 mm/min speed was chosen to represent an extreme case since higher speeds better reflect the fast solicitations during dynamic use experienced by wearable materials. Lower speeds are more accurate for assessing the impact of MWCNTs on the polymer matrix.

### 2.5. Electrical Properties

Electrical resistivity measurements for the composites were carried out using a two-point electrical resistance method with a Keithley 6487 instrument (Keithley Instruments, Cleveland, OH, USA). To evaluate the electrical resistance of the electrodes, silver ink (CI 1036, EMS, Hatfield, PA, USA) was coated on the cross-section of the opposite sides of the composite. The length of each specimen was 25 mm, the width was 10 mm, and the thickness was 0.5 mm. Therefore, the cross-section area of the silver electrodes was approximately 5 mm^2^. Subsequently, the silver-coated composites were cured at 110 °C for 20 min. Five replicates of each specimen composition were tested for statistical significance.

### 2.6. Rheological and Thermal Characterisation

Melt Flow Index (MFI) analysis was conducted to evaluate the influence of MWCNTs and TPE on the flow of the composites. This analysis was carried out using a Ceast MF20 (Instron, Norwood, MA, USA), set at a temperature of 230 °C, with a load of 2.16 kg and a pre-heating time of 120 s. To further investigate the impact of MWCNTs on the composites’ viscosity, loss, and storage modulus, oscillatory shear rheological analysis was performed. The tests were conducted on a Discovery HR 10 rheometer (TA Instruments, New Castle, DE, USA) at 230 °C. The rheological properties were assessed using a parallel stainless-steel plate geometry, with the linear viscoelastic region determined through a stress sweep at a frequency of 1 Hz. Differential Scanning Calorimetry (DSC) analysis was performed to assess the effect of MWCNTs and TPE on the composites’ melting temperature. The DSC was carried out on a DSC 214 instrument (Netzsch, Selb, Germany) over a temperature range from −40 to 300 °C at a heating rate of 10 K/min.

### 2.7. Sheath/Core Extrusion Process

Bi-component filament extrusion was conducted using two extruders, namely, “A” for TPE and “B” for the composite, with temperatures at 196 °C and 185 °C, respectively. Each extruder has a melt pump that precisely controls the volumetric throughput. Melt pump “A” has a flow rate of 0.6 cc per revolution and was set at a velocity of 6 rpm, while melt pump “B” has a flow rate of 1.2 cc per revolution and was set at 3 rpm. The transfer line temperature was maintained at 165 °C, and the spinneret at 156 °C. Spinneret pressures were 80 bar for extruder A and 119 bar for extruder B. The resulting filament consisted of a TPE sheath (extruder A) and a core comprising a 5 wt.% MWCNT/PP/TPE masterbatch, corresponding to a polymer blend of 25 wt.% TPE, 2.5 wt.% compatibilizer, and 67.5 wt.% PP. The lamination of the filaments onto commercially available knitted structures was achieved using a hot press at 130 °C, 3 bar, for 2 min.

After extrusion and textile lamination, filament characterisation was performed. Five filaments were tested; each filament’s dimensions were 25 mm in length with an internal diameter of approximately 250 µm. The electrical resistivity was measured using the same procedure and equipment described in point 2.5, where silver ink was cured on each extremity of the composite to create a silver electrode in the cross-section of opposite sides of the composites. Finally, the cross-section of the bi-component structure was observed by optical microscopy (OM) on a Leica DM300 microscope (Leica Microsystems, Wetzlar, Germany) in transmission mode.

### 2.8. Triboelectric Sensor Development

For the triboelectric sensor, a metallic mould of squares with a 1 cm side was positioned over a textile structure containing five laminated filaments. Silicone rubber (Ecoflex 00-30, Macungie, PA, USA) was poured into the mould, forming 1 mm thick square shapes on top of the textile structure. Sensor testing consisted of applying a compression force of 100 N using a Shimadzu AGX-V equipped with two compression plates, 100 mm in diameter each, at 1500 mm/min speed. The output voltage signal generated by the sensor was recorded in single electrode mode using a B&K Precision model 2194 oscilloscope (B&K Precision Corporation, Yorba Linda, CA, USA) connected with a 10 MΩ probe, as demonstrated in Video S2 (Supplementary Materials).

## 3. Results and Discussion

### 3.1. Cross-Section Morphology Analysis

**Figure 1:**
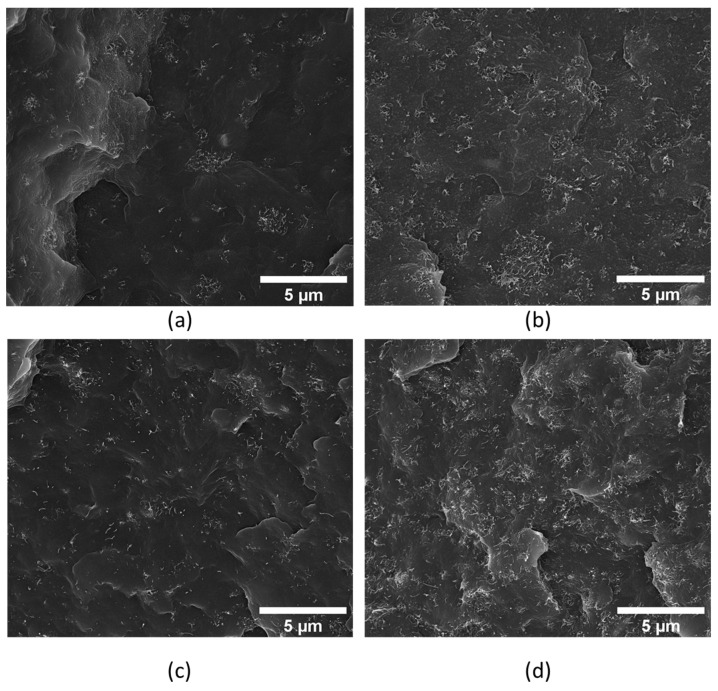
Cross-section SEM images of the produced tapes: MWCNT distribution on composites with (**a**) 2.5 wt.% MWCNT/PP; (**b**) 5 wt.% MWCNT/PP; (**c**) 5 wt.% MWCNT/PP/(25% TPE); and (**d**) 10 wt.% MWCNT/PP.

**Figure 2:**
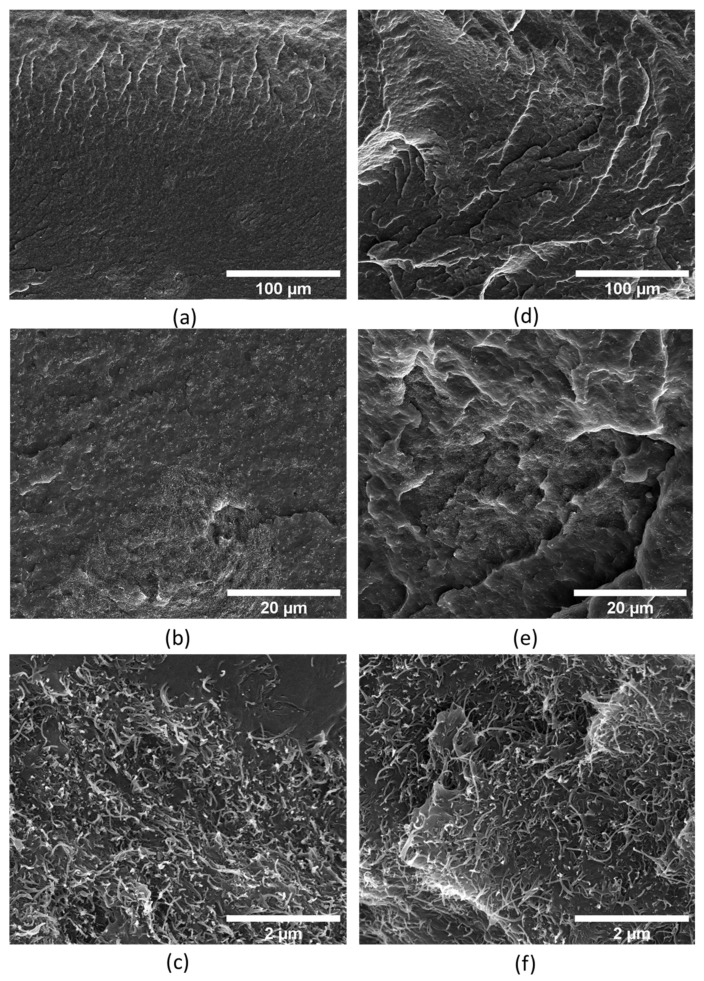
Cross−section SEM images showing MWCNT agglomerates in composites tapes with 5 wt.% MWCNT, displayed at increasing magnifications from (**a**–**c**). Images (**d**–**f**) present composites of 5 wt.% MWCNT/PP with 25 wt.% TPE at the corresponding magnifications.

The cryo-fractured surfaces of the composites prepared by twin screw extrusion were observed by SEM. Figure 1 shows the dispersed MWCNTs for the composites with increasing MWCNT concentration for composites containing 2.5, 5, and 10 wt.% MWCNTs. Figure 1c, demonstrating the PP/TPE blend, depicts good dispersion of the MWCNTs and, at this magnification, does not show signs of PP/TPE phase separation, which can also be confirmed in Figure 2.

Figure 2 illustrates the cross-section of 5 wt.% MWCNT/PP and 5 wt.% MWCNT/PP/(25 wt.% TPE) composites at different magnifications. At lower magnification, the individual MWCNTs cannot be observed. However, the greyish spots detected with approximate diameters of 50 μm and below correspond to MWCNT agglomerates. At higher magnification, these agglomerates show a higher MWCNT concentration and entanglement.

It is known that the presence of MWCNT agglomerates in the composites above a given concentration is detrimental to their mechanical properties [38]. However, it can be an advantage for the composite’s electrical conductivity. Thus, the tensile and electrical properties of the composites were measured to assess the extent of these effects.

### 3.2. Tensile Properties

**Figure 3:**
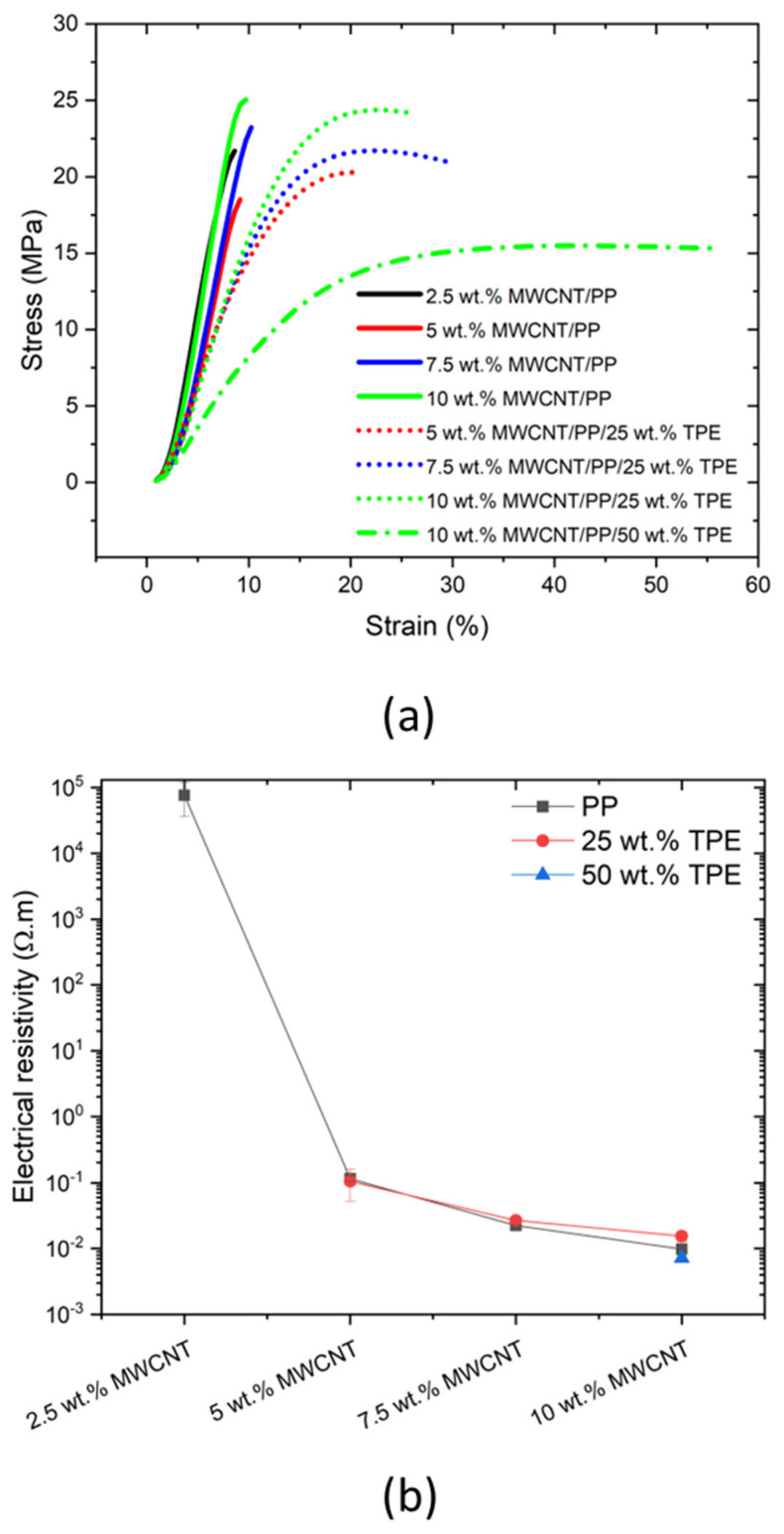
(**a**) Representative stress−strain curves of all the PP/MWCNT and MWCNT/PP/TPE composites tested at a speed of 1000 mm/min. (**b**) Electrical resistivity of the MWCNT/PP composites and their TPE blends.

The tensile properties of all the composites prepared were assessed, and representative stress–strain curves are presented in Figure 3a. Table 1 presents the tensile strength, elongation at break and Young’s modulus calculated for the composites. Figure 3a illustrates the transition from brittle to ductile behaviour achieved with the addition of TPE and the increase in concentration in the blend [39]. 

The MWCNT/PP composites display brittle behaviour, even at a low MWCNT concentration, resulting from the presence of MWCNT agglomerates and the high stiffness of the nanotubes. The composites of MWCNT/PP present tensile strength ranging from 21.1 ± 2.5 to 24.8 ± 2.1 MPa and elongation at maximum load (Rm) ranging from 9.05 ± 0.86 to 9.89 ± 0.25%. Interestingly, under the test conditions, it is observed that the tensile properties of the composites are not sensitive to composition. This is expected since nanocomposites present agglomerates with weaker bonding to the polymer which act as regions of stress concentration. At high deformation rates, there is no time for plastic deformation in the polymer to reduce stress concentrations, failure occurring due to the localised stress concentration effects. These effects are associated to the presence of nanoparticle agglomerates, which are common to all the composite concentrations.

Conversely, incorporating TPE into the MWCNT/PP composite enhances the ductility of the composites. The elongation at break significantly increases to double or triple with the addition of 25 wt.% TPE. The 10 wt.% MWCNT/PP/TPE blend with 50 wt.% of TPE has the highest ductility with average tensile strength and elongation at maximum load of 15.7 ± 0.2 MPa and 51.8 ± 5.8%, respectively. However, the rubber-like behaviour of the composites with high TPE content presents additional challenges, such as cutting the extruded tape during the composite preparation for subsequent extrusion processes. Reducing the TPE content to 25 wt.% solved these processing limitations for the three different MWCNT concentrations (5, 7.5, and 10 wt.%) prepared. The results indicate an increase in ductility for all compositions compared to the MWCNT/PP composites. The tensile strength of the blends containing 25 wt.% TPE tapes was comparable to that of MWCNT/PP composites. The elongation at break for these blends increased significantly; in the case of 7.5 wt.% MWCNT, the elongation at break more than tripled, increasing from 9.1% to 29.9%, corresponding to an approximate 330% improvement. These mechanical characteristics are particularly beneficial for laminating the tapes onto the textile substrates, warranting a flexible and seamless integration.

In summary, in the present work the aim was to test PP/MWCNT composites at deformation rates that lead to a brittle stress–strain response, and test the composite blends with TPE under these extreme conditions. This allowed to identify that using TPE leads to an increased ductile response of the composite blends, relative to the PP/MWCNT composite alone. The results demonstrate the benefits of incorporating 25 wt.% TPE to enhance the ductility of PP/MWCNT composites, which is particularly relevant for application in textiles.

### 3.3. Electrical Properties

Table 1 and Figure 3b summarise the electrical properties of the composites. The electrical percolation threshold for the MWCNT/PP composites is observed near 2.5 wt.% MWCNT, as the resistivity of pure PP is reported to be approximately 10^1^⁶ Ω·m, indicating its insulating nature. At 2.5 wt.% MWCNT, the resistivity is considerably reduced but remains relatively high due to the low MWCNT content, which is insufficient to form a large number of continuous conductive pathways for electrons to flow.

Above the percolation threshold, the electrical resistivity decreases significantly, ranging from (7.64 ± 4.03)10⁴ Ω·m to (9.83 ± 0.43)10^−3^ Ω·m for compositions with MWCNT content from 2.5 wt.% to 10 wt.%. This sharp reduction in resistivity reflects the formation of a robust conductive network built on MWCNT bridging. However, as the MWCNT content increases beyond the percolation threshold, the rate of resistivity reduction decreases, suggesting that additional MWCNTs contribute less to further enhancing conductivity once a sufficient conductive network is established [40].

Notably, the electrical properties of the PP/TPE blends are similar to those of pure PP composites. Thus, the miscibility of the polymers does not affect the electrical conductivity, meaning that the conductive network of MWCNTs is not affected by TPE blending. The morphological analysis presented in Figure 2 shows that the blend with TPE forms a homogeneous material, without phase separation, and that the MWCNTs continue to be well dispersed. The increase in MWCNT content improves conductivity consistently for both PP and its TPE blends.

Given that TPE does not influence electrical resistivity but significantly enhances mechanical flexibility, particularly at 25 wt.% TPE, the composite with 5 wt.% MWCNT/PP/25 wt.% TPE emerges as a good choice since it provides low resistivity while maintaining enhanced flexibility. Further increase in MWCNT content (e.g., to 7.5 wt.% or 10 wt.%) does not significantly decrease the electrical resistivity.

### 3.4. Rheological Characterisation

One of the objectives of this work was to prepare a flexible bicomponent filament with an electrically conductive core and an insulating sheath. This outer layer encapsulates the inner conductive composite core preventing short circuits and allowing the filament’s lamination into textile structures while protecting the integrity of the conductive core. To facilitate the lamination process, the sheath polymer was selected with a low melting temperature. Additionally, manufacturing bicomponent filaments with a core/sheath morphology requires polymers with different melt viscosity for the core and sheath. Typically, the core material should have a higher melt viscosity than the sheath material under the filament processing conditions [41]. Thus, within an acceptable range, the composite core should have a lower MFI than the sheath polymer.

**Figure 4:**
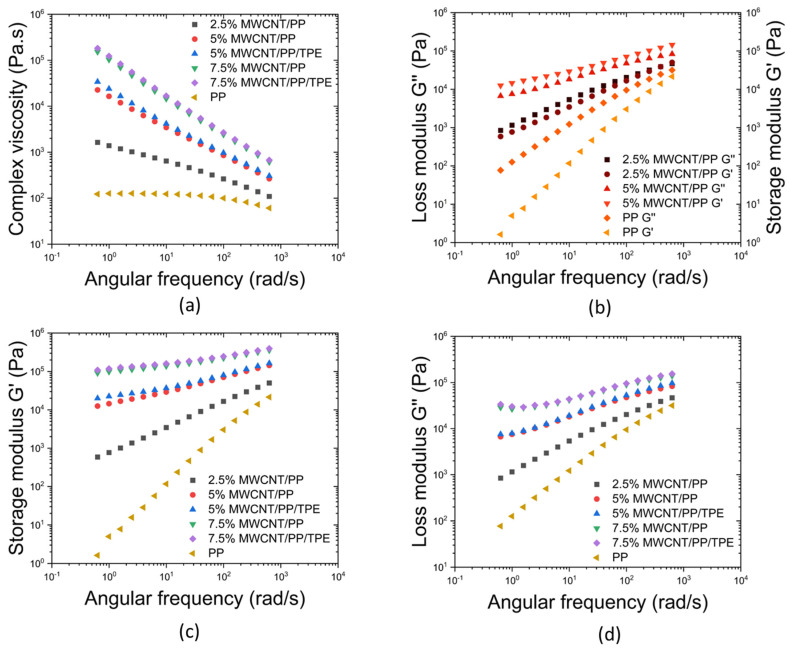
Rheology results for PP, 2.5, 5, and 7.5 wt.% MWCNT composites: (**a**) complex viscosity; (**b**) storage modulus and loss modulus for MWCNT/PP composites; (**c**) storage modulus and (**d**) loss modulus for MWCNT/PP and MWCNT/PP/TPE composites.

The MFI was measured for the polymers and composites, showing a large reduction in the MFI of the composites relative to the pure polymers. While the PP and TPE presented an MFI of 25 and 20 g/10 min, respectively, the MFI of the PP composites with 2.5 and 5 wt.% of MWCNT decreased to 15 and 5 g/10 min, respectively. Composites with higher MWCNT content did not flow under a 2.16 kg load and, thus, were not selected for the bi-component extrusion process. The MFI drop for the composites is a consequence of the formed MWCNT network hindering the polymer flow, and it is comparable with the results reported by Stanciu et al. [37]. The composites with 5 wt.% MWCNT/PP and 5 wt.% MWCNT/PP/TPE exhibit similar MFI of 5 g/10 min, thus suggesting comparable flow characteristics. These results were confirmed by oscillatory rheology, illustrated in Figure 4, showing a viscosity increase with increasing MWCNT content, particularly at low frequencies. The network formed by increasing MWCNT content creates physical barriers that restrict the polymer chains through Van der Waals interactions and entanglements. The shear-thinning behaviour of the composites, evidenced by the decrease in complex viscosity with frequency, can be attributed to the breakdown of the MWCNT network and the subsequent alignment of both the MWCNTs and polymeric chains, which reduces viscosity [36,37]. The viscosity of PP exhibits a similar trend, although less pronounced, which reinforces that the shear-thinning behaviour can also be attributed to the alignment of the polymeric chains.

Storage and loss modulus analysis reveals that TPE inclusion slightly increases the storage modulus, indicative of enhanced elasticity while maintaining a similar loss modulus. The viscoelastic behaviour of MWCNT/PP composites, especially at higher MWCNT concentrations, suggests enhanced stiffness due to the denser MWCNT network, which constrains PP chain mobility. Conversely, the 2.5 wt.% MWCNT composite, closer to the PP viscoelastic response, presents G″ slightly higher than G′, possibly indicating insufficient MWCNT dispersion, allowing for dominant viscous flow. The presence of MWCNT agglomerates is illustrated in the SEM images in Figure 1 and Figure 2.

In summary, the rheological behaviour of the composites highlights the influence of MWCNT content on viscosity and viscoelastic properties. The increase in viscosity at higher MWCNT loadings is attributed to the formation of a dense MWCNT network, which limits the mobility of polymer chains [37]. At lower MWCNT concentrations, insufficient dispersion leads to dominant viscous flow, as evidenced by the slightly higher loss modulus (G″) compared to the storage modulus (G′) and supported by SEM images showing MWCNT agglomerates. Therefore, by combining the mechanical, electrical, and rheological analyses, it was determined that the most suitable composite is 5 wt.% MWCNT/PP/25 wt.% TPE. This composition offers enhanced mechanical and electrical performance while exhibiting rheological behaviour compatible with bi-component extrusion processes. Additionally, the inclusion of 25 wt.% TPE did not negatively impact the properties compared to MWCNT/PP samples.

### 3.5. Thermal Characterisation

DSC analysis was conducted to investigate the impact of MWCNTs on the thermal properties of the PP matrix and to evaluate the thermal properties of the composite blend with TPE. Specifically, it is important to assess the melting temperature of the pure polymers, their MWCNT composites, and polymer/composite blend.

**Figure 5:**
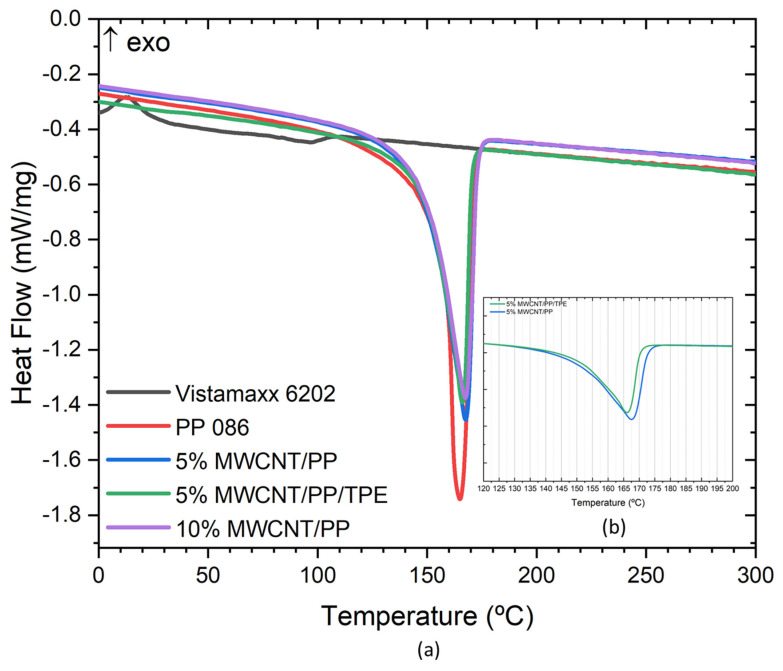
(**a**) DSC results for TPE, PP, 5 wt.% MWCNT/PP, 5 wt.% MWCNT/PP/TPE, and 10 wt.% MWCNT/PP; (**b**) representation of the melting temperature for each 5 wt.% MWCNT composite.

Two PP/MWCNT composite concentrations, 5 wt.% and 10 wt.%, were selected for this study. The influence of blending 25 wt.% of TPE with the PP/MWCNT composites was studied for the 5 wt.% MWCNT/PP/TPE blend. The thermograms are presented in Figure 5, consistent with the amorphous nature of TPE and allowing the measurement of the melting enthalpy of the PP, showing a melting temperature near 163.5 °C.

The melting temperature of the PP composites with 5 wt.% and 10 wt.% MWCNT were 166.6 °C and 166.7 °C, respectively, showing a slight increase relative to the pure polymer, however invariant with MWCNT concentration (within the concentration range studied). These results for the MWCNT/PP composites, are consistent with those reported by Stanciu et al. [37]. Blending 25 wt.% TPE with 5 wt.% MWCNT/PP composite slightly reduced the melting temperature to 165.7 °C, only 1 °C lower than the 5 wt.% MWCNT/PP alone, indicating the limited impact of MWCNTs and TPE on the crystalline structure of PP within the studied concentration range (Table 2). TPE is an amorphous polymer; thus, above the glass transition temperature (Tg) its free volume continuously increases, and the polymer decreases the viscosity until it becomes a polymer melt. TPE transforms into a melt at a lower temperature than PP, its composites, and the 5 wt.% MWCNT/PP/TPE composite blend. This is important for the lamination process of the bicomponent filaments, performed at 130 °C, thus allowing the melting of the outer TPE layer of the filament while preserving the integrity of the composite core.

The crystallinity calculation was based on the enthalpy of fusion for the different samples and the fraction of the enthalpy of fusion of pure PP in each sample. For the calculations, an enthalpy of fusion of 209 J/g for pure PP was assumed [37].

The degree of crystallisation was calculated using the following equation:

(1) $$\chi \left(\%\right)=\frac{\Delta Hm}{{∆H}^{0}\cdot \left(1-\varphi \right)}\cdot 100\%$$

where Δ*Hm* is the melting enthalpy of the sample, Δ*H*^0^ is the melting enthalpy of 100% crystalline PP (209 J/g for PP), and *φ* is the weight fraction of MWCNTs and TPE.

According to the results presented in Table 2, pure PP exhibited a crystallinity of 54.8%, and the incorporation of 5 and 10 wt.% MWCNTs did not significantly affect its crystallinity. The addition of 25 wt.% TPE to a composite with 5 wt.% MWCNT resulted in a slight increase in crystallinity, reaching 59.0%, consistent with the tendencies found by Lu et al. [42]. This increase in crystallinity can be attributed to Vistamaxx’s (TPE) ability to improve the compatibility between the composite materials. This improved compatibility facilitates better alignment and ordering of PP molecules, enhancing crystallization.

Therefore, among the tested samples, the 5 wt.% MWCNT/PP/TPE blend was identified as an adequate core material, offering a balance of thermal stability, crystallinity, and processing compatibility. With a melting temperature above 150 °C, it provides the thermal performance required for lamination processes without compromising structural integrity, making it ideal for flexible and stable textile applications. Pure TPE was selected for the outer layer of the bi-component filament due to its low melting point, ensuring compatibility with textile lamination at 130 °C while preserving the core’s structure.

### 3.6. Bi-Component Electrode Integration and Triboelectric Sensor Testing

The analysis of the composite blends provided a comprehensive evaluation of their mechanical, electrical, rheological, and thermal properties, enabling the selection of the optimal core and sheath materials for the bi-component filament extrusion process. Among the tested materials, the 5 wt.% MWCNT/PP/TPE composite blend with 25 wt.% TPE emerged as the best choice for the core. This selection was justified by the balance it offered across all critical performance metrics. Electrical resistivity results showed that increasing MWCNT content from 2.5 wt.% to 5 wt.% significantly reduced resistivity due to the formation of an electrically conductive network, while higher concentrations (e.g., 7.5 and 10 wt.%) increased viscosity, which limited the processability required for filament extrusion. In addition, the mechanical testing revealed that the inclusion of 25 wt.% TPE improved flexibility and reduced brittleness, which is critical for wearable applications under dynamic conditions. Thermal analysis further confirmed that the addition of TPE did not adversely affect the melting temperature or structural integrity of the composite core, making it compatible with the lamination process at 130 °C. The core of the filaments was therefore formed with the 5 wt.% MWCNT/PP/TPE (with 25 wt.% TPE) composite blend, while the sheath was based on pure TPE.

Subsequent electrical testing of the bi-component filaments showed similar resistivity to the composite blends. The bi-component filament and the extruded tape with the same composition presented electrical resistivities of (3.12 ± 1.06)10^−1^ Ω·m and (1.05 ± 0.53)10^−1^ Ω·m, respectively. Thus, the filament processing and the decrease in cross-section did not hinder the electrical resistivity.

**Figure 6:**
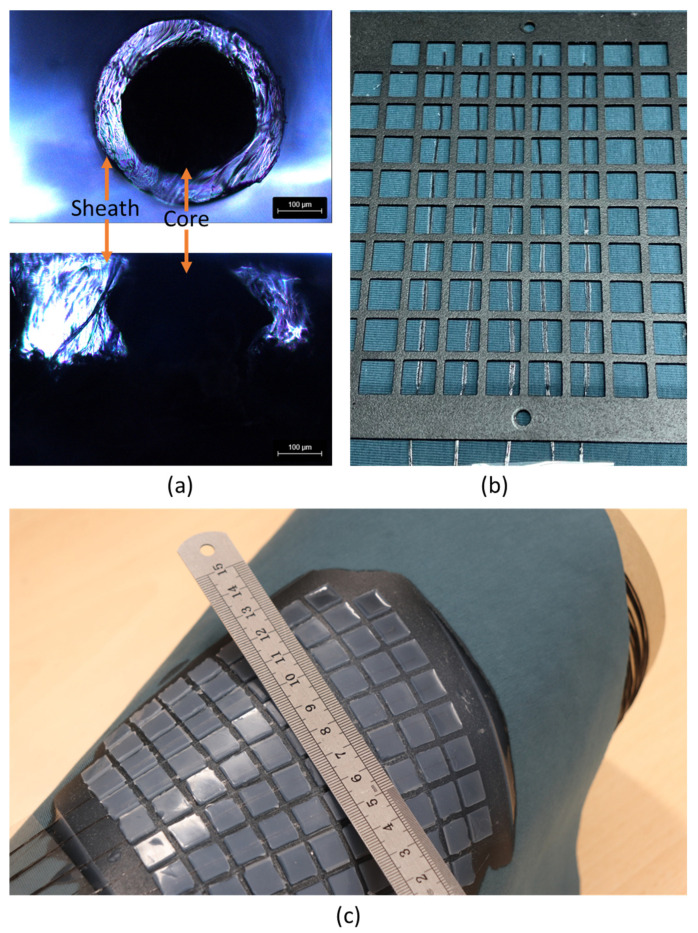
(**a**) Bi-component filament cross-section before and after hot pressing on the textile substrate. (**b**) Lamination of the filaments on a textile structure and metallic mould for the silicone rubber. (**c**) Triboelectric sensors prepared with five laminated filaments.

Then, a hydraulic hot press was used to melt the TPE sheath of the filaments against the textile structure for the smart textile integration. Since TPE softens at temperatures above 50 °C and the core melts above 160 °C, the fibre core keeps its integrity by performing the lamination process at 130 °C. In contrast, the sheath of the fibre melts and attaches to the textile surface. This can be observed in the optical images presented in Figure 6a, where the core is cylindrical and the TPE presents a rectangular shape due to the applied pressure during lamination.

Finally, to perform a preliminary demonstration of an application of these structures, a triboelectric sensor was used to detect impacts through contact and separation, as demonstrated in Video S2 in the Supplementary Materials. The bi-component sensor electrodes were integrated into the textile structure by lamination, and the textured silicone rubber was cured on top of the textile structure and electrodes, as shown in Figure 6c. Silicone rubber is commonly used in triboelectric devices for energy harvesting and sensing because of its ability to gain and donate electrons. Its high dielectric constant improves the charge storage properties and the material’s flexibility and softness optimise contact interfaces, improving electron exchange efficiency and allowing easy customisation into various shapes, suitable for several applications [21,43,44,45,46].

**Figure 7:**
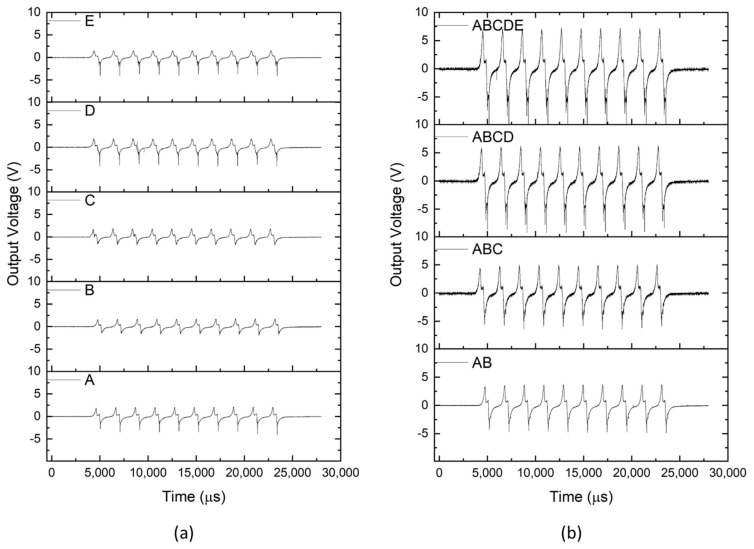
Triboelectric performance of the fabricated composite: (**a**) individual output voltage for each filament and (**b**) a combination of multiple filaments under the same applied force.

Figure 7 illustrates the impact of the sensor surface area on the output voltage of the composite. The characterisation of the triboelectric sensors revealed similar output voltage for individual filaments. When these filaments were combined, the cumulative output voltage increased, which may be attributed to the increase in sensor surface area. The 100 N of applied force from a 10 cm diameter disc ensured optimal contact with the silicone rubber surface, which is critical for consistent charge generation. The filaments were spaced 1.2 cm apart, and incorporating additional filaments expanded the effective surface area, amplifying the output voltage. The electric field resulting from the triboelectric effect facilitated the sensors’ capacity to detect impacts. Figure 7 shows that the voltage increment is residual when the number of filaments increases from 4 to 5, indicating that triboelectric coverage may be already maximised. With the silicone rubber dimensions remaining constant, three to four filaments appear sufficient to harvest the majority of the triboelectric potential between the testing disc and the silicone layer. This phenomenon was also demonstrated in the work of Xie et al. [47], who showed that increasing the mesh density beyond a certain threshold results in diminishing returns due to the saturation of effective charge collection. At this point, the system already optimally covers the triboelectric surface area and establishes a uniform electric field. A similar principle is observed in the design of electrostatic discharge (ESD) garments, where strategically spaced electrically conductive filaments efficiently dissipate static electricity without requiring full coverage [48]. This suggests that the surface area of silicone rubber interacting with the pressing element generates charges that can be effectively captured with only a few spaced filaments. Furthermore, the system’s overall capacitive properties are influenced by the addition of more filaments, each acting as a small capacitor. This can alter the charge distribution and storage, leading to a non-linear increase in voltage output.

In conclusion, the relationship between the number of filaments and triboelectric performance was primarily guided by the objective of maximising sensor coverage. The selected spacing configuration efficiently utilised the triboelectric potential of the silicone rubber surface in combination with the applied force, ensuring consistent performance and broad coverage. This approach is particularly suited to large-scale sensing applications where maximising charge generation and distribution across the sensor area is a priority. While this study focused on sensor coverage, we recognise the importance of investigating other objectives, such as improving sensor precision through the individualisation of filaments. Therefore, future work should explore how varying filament spacing affects the sensitivity and accuracy of each filament as an independent sensing element. Such an approach could provide valuable insights into the relationship between filament spacing and the spatial resolution of the sensor.

## 4. Conclusions

This study successfully demonstrates the development of thermoplastic bi-component electrodes, optimised for continuous bi-component extrusion processes, emphasising their potential for textile triboelectric impact detection sensors. The findings illustrate the ability to fine-tune the properties of thermoplastic composites to produce bi-component filaments, featuring a conductive core and an outer layer compatible with lamination processes. These filaments, fabricated through continuous extrusion, could be well-suited for large-scale manufacturing. Then, once laminated on textile substrates, they would serve as effective electrodes for triboelectric impact sensors.

The optimised composition of 5 wt.% MWCNT within a PP matrix blended with 25 wt.% TPE achieved a good balance of electrical conductivity, mechanical ductility, rheological properties, and thermal stability. The inclusion of TPE significantly enhanced the flexibility of the composites, reducing brittleness and ensuring durability under dynamic conditions such as repeated mechanical impacts.

The observed decrease in electrical resistivity with increasing MWCNT content confirmed the formation of a robust conductive network. Rheological and DSC analyses further validate the compatibility of PP/TPE for the core with the low melting point of the TPE sheath, enabling efficient lamination processes without compromising the structural integrity of the composite core. These properties make the material highly suitable for integration into textile substrates for wearable electronics, energy-harvesting systems, and safety applications.

The fabricated triboelectric sensors demonstrated reliable performance in detecting impacts, with output voltage increasing as the number of filaments increased, up to a saturation point. This highlights the need to optimise filament arrangements to balance coverage and precision effectively.

Finally, this study provides interesting results for the development of sustainable and cost-effective electrode solutions that can be used for smart textiles. Future research should focus on dynamic testing under diverse operational conditions and on conducting a detailed life-cycle assessment of these composites, to quantify their environmental impact, including energy consumption during production and recycling. Additionally, exploring methods to further enhance the recyclability of these materials and their integration into large-scale industrial processes could improve their economic and environmental viability, expanding their potential impact across diverse applications. From an economic perspective, the use of widely available and cost-effective materials such as PP and TPE enhances the scalability and affordability of the composites, making them highly attractive for commercial applications. By combining environmental advantages with economic feasibility, these composites provide a compelling solution for industries seeking to balance performance, sustainability, and cost-effectiveness.

## Figures and Tables

**Table 1:** Summary of the mechanical and electrical properties of the composites. The tensile tests were performed at 1000 mm/min.

Sample	Tensile Strength Rm (MPa)	Elongation at Break (%)	Young’s Modulus (MPa)	Electrical Resistivity (Ω·m)
2.5 wt.% MWCNT/PP	22.6 ± 0.8	9.89 ± 0.25	240.44 ± 36.98	(7.64 ± 4.03)10^4^
5 wt.% MWCNT/PP	21.1 ± 2.5	9.70 ± 0.19	226.47 ± 29.01	(1.15 ± 0.10)10^−1^
7.5 wt.% MWCNT/PP	21.1 ± 2.3	9.05 ± 0.86	230.88 ± 37.47	(2.25 ± 0.07)10^−2^
10 wt.% MWCNT/PP	24.8 ± 2.1	9.17 ± 1.10	220.53 ± 42.32	(9.83 ± 0.43)10^−3^
5 wt.% MWCNT/PP/(25% TPE)	21.1 ± 1.6	21.0 ± 0.8	153.22 ± 3.51	(1.05 ± 0.53)10^−1^
7.5 wt.% MWCNT/PP/(25% TPE)	21.8 ± 0.9	29.9 ± 1.4	110.61 ± 16.25	(2.68 ± 0.11)10^−2^
10 wt.% MWCNT/PP/(25% TPE)	24.4 ± 0.2	26.8 ± 0.9	117.51 ± 25.47	(1.55 ± 0.26)10^−2^
10 wt.% MWCNT/PP/(50% TPE)	15.7 ± 0.2	51.8 ± 5.8	68.69 ± 17.17	(7.16 ± 0.25)10^−3^

**Table 2:** Summary of the DSC results.

Sample	Onset Temperature (°C)	Peak Temperature (°C)	Enthalpy of Fusion (J/g)	Crystallinity (%)
PP	157.9	163.5	114.5	54.8
5 wt.% MWCNT/PP	148.6	166.6	108.8	54.8
5 wt.% MWCNT/PP/25 wt.% TPE	152.6	165.7	86.32	59.0
10 wt.% MWCNT/PP	148.9	166.7	103.6	55.1

## Data Availability

The original contributions presented in the study are included in the article/Supplementary Materials, further inquiries can be directed to the corresponding authors.

## Supplementary Materials

The following supporting information can be downloaded at: https://www.mdpi.com/article/10.3390/polym17020210/s1, Figure S1: Research workflow chart; Video S1: Impact testing. Video S2: Impact detection demonstration.
