# Supplementary figures and images for: Development of Thermoplastic Bi-Component Electrodes for Triboelectric Impact Detection in Smart Textile Applications
Source: Polymers (Basel). 2025 Jan 16;17(2):210. doi: 10.3390/polym17020210 (PMC11768117; doi:10.3390/polym17020210)

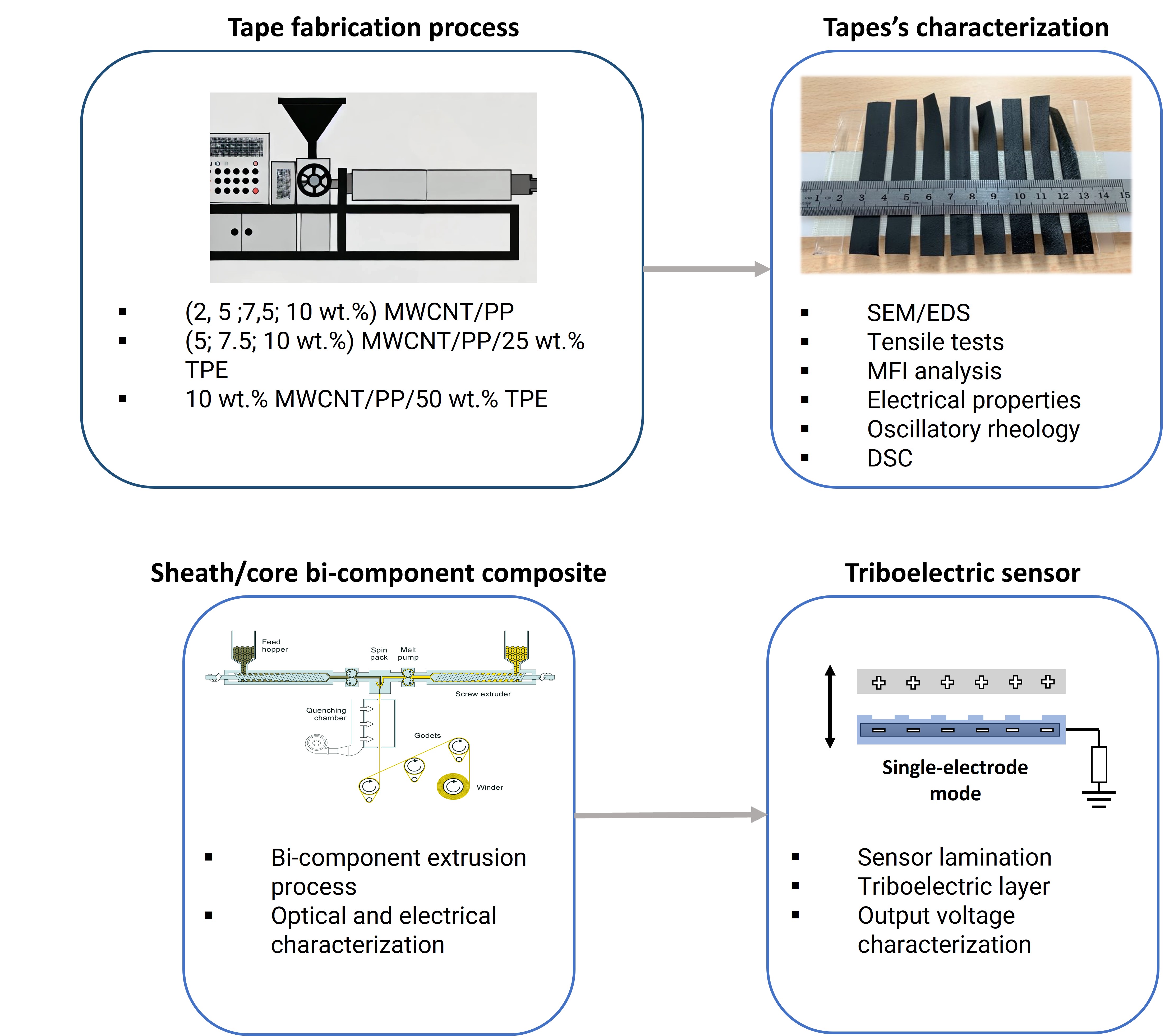

Supplement: Supplementary file 1 [file polymers-17-00210-s001.zip › S1.jpg]
